# A novel mechanism for direct real-time polymerase chain reaction that does not require DNA isolation from prokaryotic cells

**DOI:** 10.1038/srep28000

**Published:** 2016-06-23

**Authors:** Takashi Soejima, Jin-zhong Xiao, Fumiaki Abe

**Affiliations:** 1 Functional Food Ingredients, Food Ingredients & Technology Institute, Morinaga Milk Industry Co., Ltd. 5-1-83, Higashihara, Zama, Kanagawa, 252-8583, Japan; 2 Next Generation Science Institute, Morinaga Milk Industry Co., Ltd. 5-1-83, Higashihara, Zama, Kanagawa, 252-8583, Japan

## Abstract

Typically, polymerase chain reaction (PCR) is performed after DNA isolation. Real-time PCR (qPCR), also known as direct qPCR in mammalian cells with weak membranes, is a common technique using crude samples subjected to preliminary boiling to elute DNA. However, applying this methodology to prokaryotic cells, which have solid cell walls, in contrast to mammalian cells which immediately burst in water, can result in poor detection. We successfully achieved PCR elongation with the addition of 1.3 cfu of *Cronobacter muytjensii* to a newly developed direct qPCR master mix without performing any crude DNA extraction (detection limit of 1.6 × 10^0^ cfu/ml for the test sample compared with a detection limit of 1.6 × 10^3^ cfu/ml primarily for crude (boiling) or classical DNA isolation). We revealed that the chromosomal DNA retained in prokaryotic cells can function as a PCR template, similarly to the mechanism in *in situ* PCR. Elucidating this reaction mechanism may contribute to the development of an innovative master mix for direct qPCR to detect genes in a single bacterium with solid cell walls and might lead to numerous novel findings in prokaryotic genomics research.

Polymerase chain reaction (PCR) is a useful tool for the rapid detection and analysis of targeted microorganisms (particularly those that take a long time to grow or are difficult to grow artificially) in not only biochemical research samples but also food, environmental, or clinical samples^1^,^2^,^3^,^4^,^5^,^6^,^7^. Typical PCR requires time- or money-intensive DNA isolation to remove numerous PCR inhibitors^8^, although the DNA isolation may be partially conducted in an automated machine^9^.

Mammalian cells have weak cell membranes but do not have cell walls, whereas bacteria possess both^10^; thus, the chromosomal DNA of mammalian cells can easily be eluted from cells^11^. To remove the time- and money-intensive DNA isolation step, real-time PCR with crude DNA extraction (intermediate direct qPCR) through boiling has been performed with clinical samples, including hair roots. Concretely, intermediate direct qPCR has been performed by using commercially available kits, such as MightyAmp^®^ DNA Polymerase Ver.2, to suppress the function of PCR inhibitors in experimental samples (Takara-Bio, Ohtsu, Japan)^11^. The goal of this experiment was to conduct simple and rapid genomic research and routine food/environmental/clinical testing to detect DNA from nearly a single prokaryotic cell with solid cell walls. Direct qPCR with crude DNA extraction (intermediate direct qPCR) for mammalian cells is commonly applied for the detection of prokaryotic cells without any modification. This method may prevent the identification of new information regarding prokaryotic cells, as a consequence of the poor detection limit, which is at best 10^2^ cfu/ml, with average values of 10^3^ – 10^4^ cfu/ml or g. In detail, various pathogenic bacteria and fungi have been detected in faecal samples and nasopharyngeal aspirates through intermediate direct qPCR using crude DNA extraction through boiling, although the associated microorganisms in these samples, unlike mammalian cells, have solid cell walls^12^,^13^,^14^. When considering both the recovery rate of DNA extraction from cells and the contamination with PCR inhibitors originating from the tested sample matrices, crude DNA extraction methods, such as boiling, are not recommended to obtain the lower detection limit for a microorganism^15^.

Therefore, we have previously developed a series of simple and rapid real-time PCR (qPCR) techniques (direct qPCR) that do not require any DNA extraction, such as boiling, and we have applied the techniques to milk, infant formula, and blood samples^16^,^17^,^18^,^19^,^20^,^21^. The direct qPCR master mix includes a typical, real-time PCR master mix and other components, such as bovine serum albumin (BSA; Sigma, St. Louis, MO, USA), to adsorb PCR inhibitors from the test samples, a non-ionic detergent to modify the structures and dissolve the proteins of bacterial cell walls, and a calcium chelating agent^18^,^19^,^20^,^21^.

We had initially determined that this direct qPCR master mix (including the intermediate direct qPCR master mix) is more specialized than normal PCR master mix in adsorbing many of the PCR inhibitors present in samples^20^,^22^. However, the recovery rate of chromosomal DNA eluted from bacterial cells with this direct qPCR master mix remains unknown^18^,^19^,^20^,^21^. The soft membranes of mammalian cells easily burst under hyperosmolarity or hypoosmolarity, thus allowing intermediate direct qPCR elongation through the additional boiling of crude DNA extractions^23^,^24^. However, unlike mammalian cells, prokaryotic cells have solid cell walls^10^,^25^. Nonetheless, the mechanism of amplification when prokaryotic cells (and fungi) are directly added into a commercial direct qPCR master mix has not been elucidated^12^,^13^,^14^,^18^,^19^,^20^,^21^.

Indeed, there is a demand for new and precise methodologies for direct qPCR to obtain much lower detection limits for prokaryotic cells, including a more suitable direct qPCR master mix for prokaryotic cells^12^,^13^,^14^. Thus, in the present study, we present a viable method for *in situ* PCR that facilitates the physical elongation of DNA in prokaryotic cells concerning our direct qPCR. Herein, we succeeded in effectively elongating a target gene in nearly one prokaryotic cell of *Cronobacter* without performing any DNA extraction such as boiling. Because a low detection limit has never been obtained for prokaryotic cells through intermediate direct qPCR using crude (boiling) or classical approaches involving preventive DNA isolation^12^,^13^,^14^, here we report the detailed outcomes of the present study.

## Results

### PCR measurements to evaluate the disruption rate of *C. muytjensii* cell through PCR thermal cycling

To evaluate the disruption rate of *C. muytjensii* cells during PCR thermal cycling, PCR elongation was performed using a direct qPCR master mix for the pellet and supernatant obtained from *C. muytjensii* previously subjected to 50 rounds of PCR thermal cycling in physiological saline or direct qPCR components (see the Methods section) (Table 1). The direct qPCR master mix comprised of the direct qPCR components, in which the volume of sterile water (SW) was reduced to 2.7 μl, and the typical PCR master mix including Taq, specific (*omp*A) primers for *C. muytjensii*, and SYBR Green I.

The effects of matrices with direct qPCR components before and after PCR thermal cycling on PCR elongation were evaluated, to accurately evaluate Table 1 (Table 2). With respect to the samples amplified without thermal cycling (hereafter termed non-heated), we used *C. muytjensii* in physiological saline. A comparison of the Ct values for the suspension (corresponding to Pellet in Table 1) and Supernatant (Table 1) obtained through centrifugation revealed that the Ct values for the Pellet group were approximately 5 Ct units lower than those of the Supernatant group. In contrast, given the experimentally determined PCR efficiency (average of 2.1 nearing theoretical value of 2.0) calculated in the range of 2.5 × 10^3^ to 2.5 × 10^6^ cfu/PCR for *C. muytjensii* in saline (Table 2), the number of PCR amplicons increased approximately 10^1^-fold (2.1^3.2^-fold) after 3.2 cycles of PCR, and the Pellet group was approximately 5 Ct units lower than the Supernatant group, thus suggesting that the DNA amount in the Pellet group was 2.1^5^-fold (1.6 log_10_-fold) higher than that in the Supernatant group^26^. In relation to the *C. muytjensii* suspension with direct qPCR components, the Ct values for the Pellet group were 8.2 to 8.3 Ct units lower than those of the Supernatant group, regardless of PCR thermal cycling parameters (Table 1). Concerning the inhibitory effect of direct qPCR components compared with saline, no significant differences were observed before or after PCR thermal cycling between the Pellet and Supernatant groups (Table 2). The PCR efficiencies were calculated in a range from 2.5 × 10^3^ to 2.5 × 10^6^ cfu/PCR for *C. muytjensii* in the Pellet and Supernatant groups with and without PCR thermal cycling, and the efficiency values were 2.2 (Pellet with non-heated direct qPCR components), 2.1 (Supernatant with non-heated components), 2.1 (Pellet with heated components), and 2.1 (Supernatant with heated components). These calculations suggest that the DNA amounts in the Pellet groups could be 2.1^8.2^– 2.2^8.3^-fold (2.6 – 2.8 log_10_-fold) higher than those in the Supernatant groups, regardless of PCR thermal cycling (Table 2).

**Figure 1:**
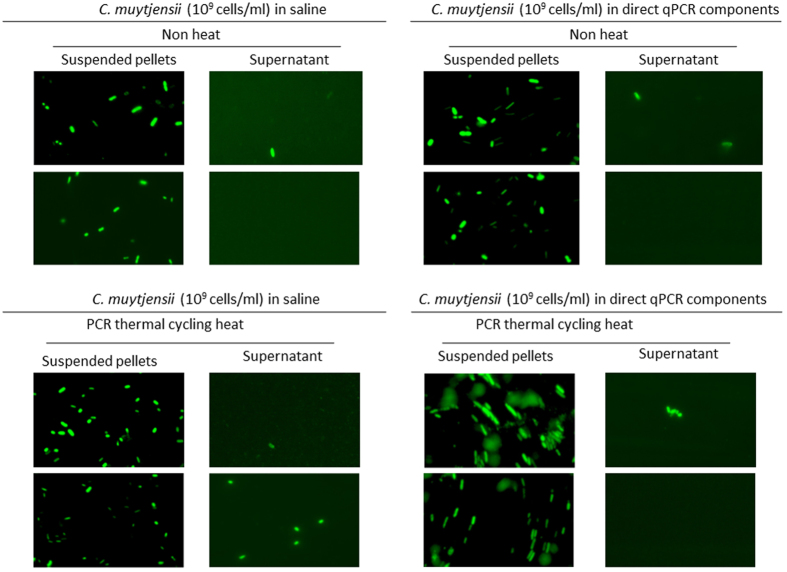
Fluorescence microscopy images (SYTO9) of *C. muytjensii* in physiological saline or the direct qPCR components before or after PCR thermal cycling.

In addition, the Ct values of the Pellet and Supernatant groups for *C. muytjensii* suspended in the direct qPCR components after PCR thermal cycling were by 6.6 to 6.7 higher than those of the Pellet and Supernatant groups, prior to PCR thermal cycling (rows 5 and 6, and rows 7 and 8 in Table 1). These significantly higher Ct values did not result from the inhibitory effects of the sample matrices of the direct qPCR components after PCR thermal cycling, because there were no significant differences in the PCR elongation between saline and direct qPCR components before and after PCR thermal cycling (Table 2). However, the originally transparent direct qPCR components became cloudy after 50 cycles of PCR thermal cycling (see cloudy particles of the second half and right-side image in Fig. 1). When *C. muytjensi* was suspended in the cloudy direct qPCR components, and this was followed by PCR in newly prepared direct qPCR master mix, no inhibitory effects were observed (Table 2). This result suggests that *C. muytjensii* exposed to gradually producing clouded compartments of the direct qPCR components could not immediately function as a PCR template in successive direct qPCR, reflecting adsorption and/or folding of the cloudy components.

To estimate the number of non-heated *C. muytjensii cells* suspended in saline that were recovered in the Supernatant through centrifugation, live bacterial counts were obtained after growth on standard plate count agar medium (SPC agar: Eiken, Tokyo, Japan) for the Supernatant and Pellet groups (Table 1). The live *C. muytjensii* cell counts for the Pellet and Supernatant groups were 7.6 ± 0.19 and 5.7 ± 0.20 log_10_ cfu/ml, respectively. This result shows that the live count for the Pellet group was approximately 1.9 log_10_ cfu/ml unit higher (by 1 << 2 log_10_ cfu/ml unit order for raw counting data) than that of the Supernatant group. Regarding the non-heated *C. muytjensii* suspension in physiological saline, the PCR data were highly consistent with that of the SPC counts.

### Morphological analysis of *C. muytjensii* cells in physiological saline or direct qPCR components after PCR thermal cycling

**Figure 2:**
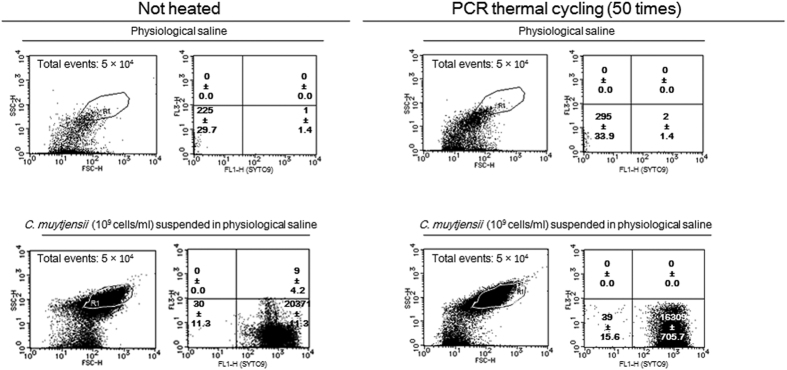
FCM assay with SYTO9 of *C. muytjensii* in physiological saline before or after PCR thermal cycling. The particles in the gate R1 in an FSC-H-SSC-H plot, were plotted on quadrants in the SYTO9-FL3-H graph. The FCM measurements were completed in duplicate, and the particle numbers of quadrants are presented as the means ± SD (n = 2).

**Figure 3:**
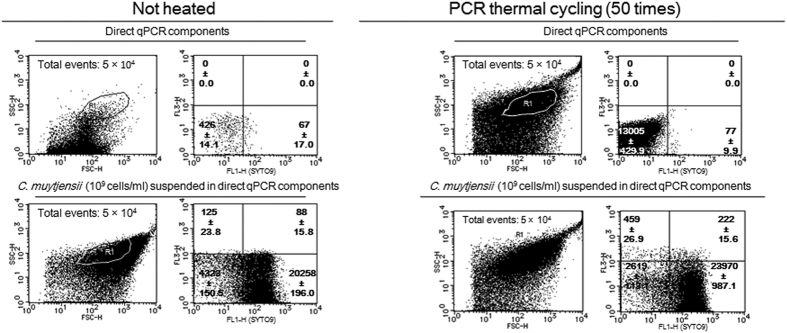
FCM assay with SYTO9 for *C. muytjensii* in the direct qPCR components before or after PCR thermal cycling. The particles in the gate R1 in an FSC-H-SSC-H plot were plotted on quadrants in the SYTO9-FL3-H graph. The FCM measurements were completed in duplicate, and the particle numbers of the quadrants are presented as the means ± SD (n = 2).

Fluorescence microscopy and flow cytometry (FCM) measurements using SYTO9 nucleic acid staining (Molecular Probes, Inc., Eugene, OR, USA), which penetrates into both intact and compromised cell walls/inner membranes of *C. muytjensii*, were performed to determine whether *C. muytjensii* retained their morphology after PCR thermal cycling (Figs 1, 2, 3).

As observed in the green fluorescent bacteria (Fig. 1), significant differences between the suspended pellets (corresponding to Pellet in Table 1) and supernatant (corresponding to Supernatant in Table 1) occurred with and without PCR thermal cycling, regardless of the different suspension matrices (saline and the direct qPCR components). In both matrices, after PCR thermal cycling, the green fluorescent bacteria retained the morphology of bacterial cells with chromosomal DNA, and the numbers were at the same level as that before PCR cycling for the suspended pellets (Fig. 1).

The FCM assays for *C. muytjensii* suspended in saline before and after PCR thermal cycling are shown in Fig. 2. Similarly, the FCM assays for *C. muytjensii* suspended in the direct qPCR components before and after thermal cycling are presented in Fig. 3. The plot number in the SYTO9 quadrant (+)/FL3-H(−) for the *C. muytjensii* suspension in saline after PCR thermal cycling was approximately 80% of that before thermal cycling (Fig. 2). In contrast, relative to the comparison between *C. muytjensii* suspended in the direct qPCR components before and after PCR thermal cycling, the plot number of SYTO9 (+)/FL3-H (−) for the *C. muytjensii* after PCR thermal cycling was the same as that of the associated cell suspension before PCR cycling (Fig. 3), suggesting that the *C. muytjensii* in the direct qPCR components was not degraded through PCR thermal cycling.

### The degree of PCR elongation and the FCM assay for *C. muytjensii* subjected to fixation before, during, and after direct qPCR measurements

**Figure 4:**
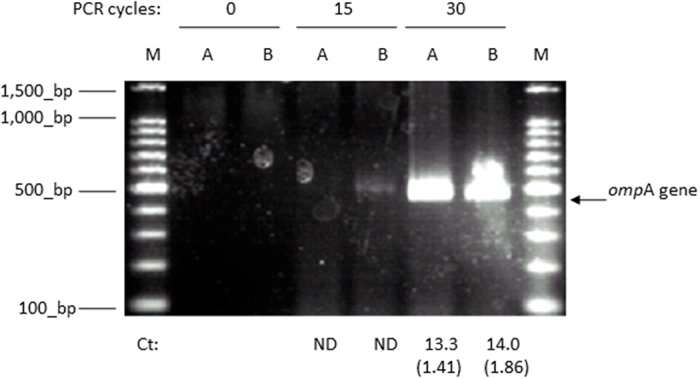
Gel-electrophoresis of the supernatant resulting from the direct qPCR master mix containing the fixed *C. muytjensii* cells before, during, or after direct qPCR. A 2.0% agarose gel was used. M: 100 bp DNA ladder; A: fixation with 4% paraformaldehyde (4% PFA); B: fixation with methanol/acetic acid (3/1). Under the gel image, the Ct values of the associated direct qPCR are presented as the means ± SD in parenthesis (n = 2); ND: no elongation.

To determine whether PCR elongation through direct qPCR is similar to that in *in situ* PCR, we performed direct qPCR elongation (0, 15, or 30 cycles) on *C. muytjensii* with preliminary fixation A (4% paraformaldehyde, PFA) or B (methanol/acetic acid = 3/1) (Fig. 4) and also performed FCM assays (SYTO9 staining) for the direct qPCR products at 0, 15, or 30 cycles on fixed cells (Tables 3 and 4). Although the chromosomal DNA was retained in *C. muytjensii* cells after fixation, the gel-electrophoresed bands for both supernatants obtained through centrifugation after direct qPCR with 30 cycles were positive (Fig. 4). In contrast, compared with the quadrant numbers of the SYTO9 (+)/FL3-H (−) in the FCM plots for the pellets obtained through centrifugation of the direct qPCR products at 0, 15, or 30 cycles, there were few significant differences between either fixation method (Fig. 4). These results suggest that a large part of the chromosomal DNA from the fixed cells was retained in the cells, but that PCR elongations took place; as a consequence, the elongated amplicons (*omp*A of 469 bp) were released from the bacterial cells into the external direct qPCR master mix.

### Direct qPCR is similar to *in situ* PCR

**Figure 5:**
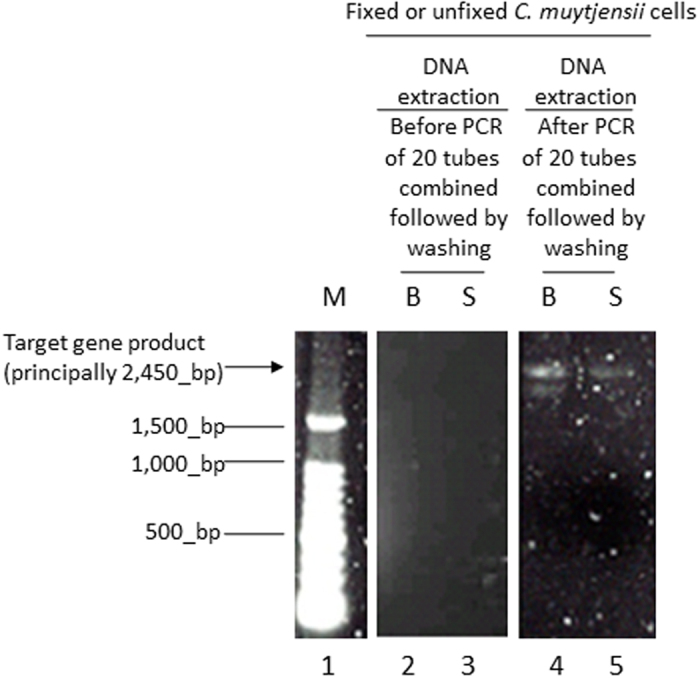
Electrophoresis images of DNA extracted from the *C. muytjensii* cells with or without fixation followed by direct qPCR producing long DNA amplicons. M: 100 bp DNA ladder; B: fixation B (methanol/acetic acid = 3/1); S: no fixation (use of physiological saline); Target gene length: principally 2,451 bp of the 16 S to 23 S rRNA gene.

When PCR elongation and *in situ* PCR were conducted in the *C. muytjensii* cells, the typically short DNA amplicons (*i.e.*, *omp*A of 469 bp) were eluted from the bacterial cells (Fig. 4); thus, to ascertain that the PCR amplicons resulted from *in situ* PCR in bacterial cells, long DNA amplifications for *C. muytjensii* with no fixation (labelled as S in Fig. 5) or fixation B (methanol/acetic acid = 3/1 labelled as B in Fig. 5) were performed. Figure 5 indicates that long DNA amplicons (16S–23S rRNA gene; approximately 2,450 bp) were retained in bacterial cells. Next, DNA extraction was performed using the pellets obtained through centrifugation of the PCR products (followed by combining 20 PCR tubes) after direct qPCR. As presented in lanes 4 and 5 of Fig. 5, the presence of long DNA PCR amplicons (2,450 bp) was confirmed. As a control, DNA extractions were performed on the pellets obtained through centrifugation of the PCR reaction mixture (combining 20 PCR tubes) prior to direct qPCR, and no bands were detected (lanes 2 and 3 in Fig. 5). These results suggest that the long DNA amplicons, at least in part, were retained in *C. muytjensii* cells.

### Comparison of the detection limits for *Cronobacter* cells after direct qPCR or qPCR following crude DNA extraction or DNA purification

We obtained 1 ml of 1.6 × 10^0^–1.6 × 10^5^ cfu/ml of the *C. sakazakii* and *C. muytjensii* suspensions in SW, on which we performed direct qPCR amplification or qPCR elongation after crude DNA extraction (boil for 10 min) or DNA purification using the FastPure DNA Kit (Takara Bio). The results are presented in Table 5. As a control, qPCR standards of the serially diluted purified chromosomal DNA are also shown in Table 5. The Ct values for the DNA (qPCR standards) and Cell (direct qPCR using *Cronobacter* cells) groups in Table 5 were approximately the same between the relevant cell numbers of *C. sakazakii* or *C. muytjensii*, suggesting that nearly 100% of the chromosomal DNA retained in the cells was available for direct qPCR elongation. The following detection limis for each method (Table 5) were observed: 1.6 × 10^0^ cfu/ml (corresponding to 1.3 × 10^0^ cfu/PCR) for DNA (qPCR standards) and Cell (direct qPCR), 1.6 × 10^2^ (*C. sakazakii*) or 1.6 × 10^3^ cfu/ml (*C. muytjensii*) for the Clean-up methods, and 1.6 × 10^3^ cfu/ml (both *Cronobacter* species) for the Boil methods. Comparison of the detection limits for *Cronobacter* revealed that the direct qPCR system utilizing *in situ* PCR for prokaryotic cells was superior to qPCR following crude or purified DNA extraction, indicating a significant advantage in the robust detection of nearly one singular bacterial cell.

## Discussion

When the DNA or RNA recovery from bacteria is nearly 100%, using crude extraction techniques, such as boiling, before intermediate direct qPCR could succeed in detecting a single cell. However, as previously discussed, the detection limit for prokaryotic cells and fungi, through intermediate direct qPCR using boiling is at best 10^2^ cfu/ml, with an average of 10^3^ to 10^4^ cfu/ml or g^12^,^13^,^14^. Therefore, we used direct qPCR, which does not depend on crude DNA extractions. Initially, when *C. muytjensii* cells were directly added to the direct qPCR master mix (see the Methods section), we incorrectly presumed that the DNA elution from these microorganisms into the external direct qPCR master mix might be induced during PCR thermal cycling. Briefly, we thought that repeated increases in the temperature would cause physical injury to the *C. muytjensii*; thus, thermal cycling would correspond to the aforementioned crude DNA extraction methods. As described in the Methods section, the direct qPCR master mix used in the present study comprises a 2-fold higher concentration of DNA polymerase and an approximately 2.4-fold higher concentration of Mg^2+^ and primers than the commercial MightyAmp direct qPCR master mix (Takara-Bio). Furthermore, our new mix has a unique combination of the PCR additives, including BSA, lysozyme, non-ionic detergent of polyoxyethylene (10) cetyl ether (Brij56), and trisodium citrate dehydrate (TSC) (for details, see Supplementary Table 1). However, we had initially added a higher concentration of the aforementioned components to accelerate PCR elongation at the highest possible reactivity under many the PCR inhibitors present in milk and blood samples^15^.

If DNA from the *Cronobacter* cells were indeed eluted into the external direct qPCR master mix during PCR thermal cycling, the chromosomal DNA would be mainly distributed in the supernatant but not in the bacterial pellets obtained through centrifugation after direct qPCR. However, the comparison of Ct values in rows 7 and 8 of Table 1 revealed that the Ct of the Pellet was 8.2 units lower than the Ct of the Supernatant; thus, the DNA could be distributed in the pellets at a ratio of more than 100-fold higher than that in the supernatant, considering the actual PCR efficiency experimentally obtained from Table 2. Therefore, we discarded the previous hypothesis and proposed that direct qPCR without crude DNA extraction might use the same reaction mechanism as that of *in situ* PCR^27^ because the DNA elution from bacterial cells into the direct qPCR master mix did not occur (rows 7 and 8 in Table 1). *In situ* PCR is a biotechnological approach that facilitates PCR elongation in prokaryotic and eukaryotic cells. Target cells need exposure to a low dose of protease, brief heating period or electron irradiation because typical *in situ* PCR requires that the cell walls/inner membranes be compromised to allow effective penetration of all the *in situ* PCR master mix components, including DNA polymerase, into the targeted cells^27^. Furthermore, each PCR master mix component of the *in situ* PCR must be significantly higher in concentration than the components in a typical PCR to facilitate the penetration of all master mix components into the cells^27^. Particularly, the DNA polymerase concentration for *in situ* PCR should ideally be 10-fold higher than that in typical PCR, and the Mg^2+^ concentration must be increased 2-fold. As previously mentioned, the components in the direct qPCR master mix used in the present study meet the requirements for *in situ* PCR.

According to Figs 1 and 3, nearly 100% of *C. muytjensii* cells suspended in the direct qPCR components maintained normal morphology and retained chromosomal DNA after PCR thermal cycling. As previously estimated, direct qPCR utilized the chromosomal DNA retained in *C. muytjensii* cells as a PCR template. Therefore, to ascertain that the mechanism of direct qPCR is similar to that of *in situ* PCR, we determined that the PCR elongation proceeded in fixed *C. muytjensii* cells whose chromosomal DNA was bound to membrane proteins originating from the bacterial cells; and then, its genome DNA was retained in the cells in the direct qPCR system (Fig. 4). Furthermore, according to the FCM assay and fluorescence microscopy analysis, increased thermal cycling did not accelerate the disruption of *C. muytjensii* cells in direct qPCR (Tables 3 and 4, Figs 1 and 3). Thus, the chromosomal DNA inside *C. muytjensii* cells emitted green fluorescence derived from SYTO9, which intercalated with the double-stranded chromosomal DNA (Tables 3 and 4, Figs 1 and 3).

Additionally, the confirmation of green fluorescent bacteria in the supernatant under the four different conditions revealed the presence of *C. muytjensii* having chromosomal DNA in the supernatant obtained through centrifugation (Fig. 1), at a level less than 10% of that in the relevant suspended pellets (Fig. 1), suggesting that the DNA elongation of Supernatant samples shown in Table 1 could result from the aforementioned retained *C. muytjensii* cells but not the chromosomal DNA eluted into the Supernatant.

Therefore, we demonstrated at an initial stage of the direct qPCR that the chromosomal DNA retained in microorganisms could function as a PCR template comparable to that used in *in situ* PCR.

As another approach to indicate that the direct qPCR for prokaryotic cells was comparable to *in situ* PCR, we revealed the presence of long amplicons (primarily 2,450 bp of the 16 S to 23 S rRNA gene) in *C. muytjensii* cells (lanes 4 and 5 in Fig. 5). This result confirmed that the PCR elongation was comparable to that of *in situ* PCR.

Next, a comparison of the Ct values of the DNA (qPCR standards) and Cell (direct qPCR) samples shown in Table 5 revealed that the chromosomal DNA of nearly 100% of the *Cronobacter* cells could be utilized as the PCR template in direct qPCR. The direct qPCR master mix used in the present study, which was designed for *in situ* PCR for prokaryotic cells, is responsible for the low detection limit of 1.6 × 10^0^ cfu/ml (1.3 × 10^0^ cfu/PCR) for direct qPCR (Cell in Table 5). Furthermore, a low detection limit for *Cronobacter* cells with direct qPCR are never obtained through qPCR using purified or crudely extracted DNA (Clean-up or Boil in Table 5). Therefore, the direct qPCR master mix developed in the present study facilitated the detection of bacterial cells of interest in typical research samples in which the levels of these cells was below the detection limit of typical PCR using various DNA purification methods or crude extraction.

Moreover, concerning the discrimination of live from dead microorganisms of interest, the potential use of ethidium monoazide (EMA) or propidium monoazide (PMA) combined with direct qPCR is feasible (Supplementary Table 1)^16^,^28^,^29^.

## Conclusion

For routine use, we propose that the elucidation of the mechanism underlying this methodology (which is similar to that of *in situ* PCR) will lead to the development of a further improved direct qPCR master mix for prokaryotic cells that does not require manual DNA isolation or an automated machine for DNA purification. Because the detection of cells of interest using direct qPCR reaches a level of nearly 1 cell per ml of the test samples, this technology could supply numerous novel findings for genome research and food/environmental/clinical testing for prokaryotic cells. This mechanism could also significantly impact manufacturers that have previously developed a direct qPCR master mix.

## Methods

### Strains and culture methods

*C. muytjensii* ATCC51329 and *C. sakazakii* ATCC29544 were cultured at 37 °C for 16 h using Brain Heart Infusion Broth (Eiken Chemical Co., Ltd., Tokyo, Japan). An aliquot of 5 ml of the culture was placed in a 15-ml Falcon tube (Becton Dickinson Labware, Franklin Lakes, NJ, USA) and subjected to refrigerated centrifugation at 3,000 × *g* for 10 min at 4 °C. The supernatant was removed, and 5 ml of physiological saline was subsequently added to the pellet to prepare a stock live cell suspension of *Cronobacter*. The live cell suspension was appropriately diluted with physiological saline as in the test sample. Independently, 1 ml of the aforementioned stock of live cell suspension was placed in a 1.5-ml volume microtube (Eppendorf, Hamburg, Germany), and the tube was subsequently immersed in a boiling water bath for 50 sec and immediately quenched. To generate a stock of heat-killed (dead) cell suspension, we confirmed that the bacteria did not thereafter form any colonies on a standard plate count agar medium (SPC agar: Eiken). The viable cell count of *C. muytjensii* or *C. sakazakii* live cell suspension was conducted on SPC agar medium, and measurements of turbidity were simultaneously performed at a wavelength of 600 nm using a spectrophotometer U-2800 A (Hitachi, Tokyo, Japan) to determine the relationship between the live cell count and turbidity.

### Direct qPCR measurement of pellets and supernatant obtained through centrifugation of *C. muytjensii* cell suspension after PCR thermal cycling

The direct qPCR components comprised 0.5 μl of 500 μg/ml lysozyme, 6.25 μl 4% Brij56, 2.5 μl 2% BSA, 0.1 μl 250 mM TSC, 0.1 μl 750 mM MgCl_2_, 0.1 μl 320 × SYBR Green I, and 15.45 μl of SW (total 25 μl). Each *C. muytjensii* suspension (physiological saline or direct qPCR components) was divided into 25-μl aliquots and placed into 200-μl PCR tubes. The 25-μl portions were subjected to PCR thermal cycling involving 50 cycles at 95 °C for 15 sec, 60 °C for 20 sec, and 72 °C for 30 sec and were subsequently recombined into one tube (total 0.25 ml). A total of 0.25 ml of heat-treated *C. muytjensii* suspension was centrifuged at 10,000 × *g* for 5 min at 4 °C, and the supernatant was transferred to a new microtube.

Freshly prepared saline or direct qPCR components (0.25 ml) were added to the obtained pellets, and an aliquot of 2.5 μl of the re-suspension (0.25 ml) was added to 12.25 μl of fresh direct qPCR components (the volume of SW was changed from the aforementioned 15.45 to 2.7 μl; hereafter unless otherwise mentioned, the volume of SW was 15.45 μl), followed by the addition of 12.75 μl of the typical PCR master mix described below (that is, 25 μl of the direct qPCR master mix), corresponding to “Pellet (suspension)” in Table 1. The specific primers for *C. muytjensii, omp*A_F (the forward primer for *omp*A gene elongation; 5′-GGATTTAACCGTGAACTTTTCC-3′) and *omp*A_R (the reverse primer for *omp*A gene elongation; 5′-CGCCAGCGATGTTAGAAGA-3′) were used, resulting in the amplification of a 469 bp product^30^. The typical PCR master mix comprised 2 μl of 10 pmol/μl *omp*A_F, 2 μl of 10 pmol/μl *omp*A_R, 0.25 μl of 5U/μl Ex-Taq (Takara-Bio, Ohtsu, Japan), 2.5 μl of 10 × Ex-Taq Buffer (Takara-Bio), 2 μl of 2 mM dNTP mixture, and 4 μl of 10 × SYBR Green I Nucleic Acid Gel Stain (10,000 × concentrate in DMSO, Lonza Rockland, Inc., Rockland, ME, USA).

Approximately 12.25 μl of the direct qPCR components (2.7 μl of SW ver.) and 12.75 μl of the typical PCR master mix (that is, direct qPCR master mix) were also added to an aliquot of 2.5 μl of the supernatant following the centrifugation of the aforementioned *C. muytjensii* suspension, corresponding to “Supernatant” in Table 1. The thermal cycle program included 1 cycle at 4 °C for 3 min, 50 cycles at 95 °C for 15 sec, 60 °C for 20 sec, and 72 °C for 30 sec, followed by 1 cycle at 95 °C for 3 min and a successive melt analysis of the PCR amplicons. Additionally, the Ct value in qPCR represents the first PCR cycle at which the fluorescence value resulting from PCR elongation is above the threshold, and subsequently, the Ct value decreases with increasing DNA template concentration in the PCR tube.

### Influence of direct qPCR components before and after thermal cycling on the PCR elongation in terms of PCR inhibitors

Ten 25-μl portions of the direct qPCR components were subjected to 50 cycles of PCR thermal cycling and combined with one portion (0.25 ml) to prepare the direct qPCR components (matrices) heated through thermal cycling. Concerning the pellet or supernatant obtained through centrifugation at 10,000 × *g* for 5 min at 4 °C to its one portion, the former was re-suspended in 0.25 ml of freshly prepared direct qPCR components, and then the latter was used as it is for sample matrices (corresponding to Pellet or Supernatant of Heat following PCR thermal cycling in Table 2). Likewise, the same experimental procedure was performed to prepare sample matrices regarding ten 25-μl portions of the direct qPCR components without PCR thermal cycling (corresponding to Pellet or Supernatant of No heat in Table 2).

Subsequently, the *C. muytjensii* cells were cultured overnight, washed, and suspended in physiological saline or the direct qPCR components (corresponding to 2 kinds of Pellet or Supernatant matrices in Table 2) with or without PCR thermal cycling, at a concentration of 2.5 × 10^6^ to 10^9^ cfu/ml. To an aliquot of 2.5 μl of each bacterial suspension, 12.25 μl of direct qPCR components (2.7 μl of SW ver.) and 12.75 μl of the typical PCR master mix (including *C. muytjensii omp*A forward and reverse primers) were added to perform the PCR.

### Examination of *C. muytjensii* cell morphology in physiological saline or direct qPCR components after the PCR thermal cycling

*C. muytjensii* cells were cultured overnight and washed, and the bacterial pellet was suspended in physiological saline or direct qPCR components (0.25 ml) at a concentration of 10^9^ cfu/ml. Each suspension was divided into 25 μl aliquots and placed into a 200-μl PCR tube followed by PCR thermal cycling (50 cycles at 95 °C for 15 sec, 60 °C for 20 sec, and 72 °C for 30 sec). The contents were combined (total 0.25 ml). Each combined 0.25-ml bacterial suspension was halved; one half was retained in the original state, and the other half was subjected to centrifugation at 10,000 × *g* for 5 min at 4 °C, and the supernatant was recovered. To 0.125 ml of each of the aforementioned bacterial suspensions and supernatants, SYTO9 (Molecular Probes) was added at a ratio of 1.5 μl/ml and incubated at 4 °C for 15 min under a safelight. Subsequently, an aliquot of 2.5 μl was placed onto a glass slide, covered with a cover slip, and examined on an AxiosKop2 motplus fluorescence/stereoscopic microscope (LEJ Leistungselektronik Jena GmbH, Jena, Deutschland) to determine whether the bacterial cell emitted λ_max_530 nm green fluorescence with an argon laser and a 488 nm excitation.

Similarly, the *C. muytjensii* suspension (10^9^ cfu/ml; finally 0.25 ml) in 25-μl aliquots with physiological saline or direct qPCR components, was subjected to PCR thermal cycling, including 50 cycles or no PCR cycles, and the divided portions were pooled, generating a total of 0.25 ml. Each suspension (0.25 ml) was centrifuged at 10,000 × *g* for 5 min at 4 °C followed by the removal of the supernatant; the resulting bacterial pellet was suspended with 0.25 ml of physiological saline. Similarly, SYTO9 staining was performed for the measurement of FCM; the FACSCalibur flow cytometer (Becton Dickinson, San Jose, CA, USA) and an argon laser of 488 nm were used to determine the bacterial cell plots through FSC (forward scattering light) and SSC (side scattering light). When the SYTO9 staining agent intercalated into the intracellular chromosomal DNA, the FL1 filter (of which λ_max_ is 530 nm by excitation with argon laser irradiation) detected the green fluorescence of the SYTO9, and the FL1 signal was plotted. Although no nuclear staining agents based on propidium iodide (PI) were used, the red fluorescence was collected using the FL3 filter as a reference. The detailed measurement conditions of FCM are presented below:

p1 of Parameter: FSC of Detection, E02 of Voltage, log of Mode; p2 of Parameter: SSC of Detection, 458 of Voltage, log of Mode; p3 of Parameter: FL1 of Detection, 820 of Voltage, log of Mode; p4 of Parameter: FL2 of Detection, 643 of Voltage, log of Mode; p5 of Parameter: FL3 of Detection, 643 of Voltage, log of Mode; p6 of Parameter: FL1-A of Detection, 1.00 of Amp-Gain, lin of Mode; p7 of Parameter: FL1-H of Detection, 1.00 of Amp-Gain; lin of Mode. Compensation was below: FL1 – 47.2 FL2; FL2 – 37.7 FL 1; FL2 – 44.7 FL3; FL 3–36.5 FL2. Threshold: Primary parameter of FSC-H 0. Total events in FSC-SSC plots: 5 × 10^4^. Hereafter, all the FCM measurements followed the conditions described in this section.

### Direct qPCR measurements of the *C. muytjensii* cells subjected to fixation and FCM to determine morphology retention after PCR thermal cycling

Two 500-μl portions of the *C. muytjensii* cells (9.3 × 10^8^ cfu/ml) were cultured overnight and centrifuged at 3,000 × *g* for 10 min at 4 °C. The supernatant was removed, and 500 μl of fixation A (4% paraformaldehyde, PFA) or fixation B (methanol/acetic acid = 3/1) was subsequently added to the pellet. The mixture was incubated overnight at 4 °C to cross-link the chromosomal DNA in the *C. muytjensii* cells and cell wall proteins to fix the DNA in the bacterial cells. Next, the cells were washed 3 times with 500 μl of physiological saline and were subsequently suspended in 250 μl of physiological saline. The recovery rate of bacterial cells to pellet after each centrifugation for washing was experimentally determined to be 80% (legend of Table 5), and centrifugation was conducted four times. Thus, the concentration of the *C. muytjensii* suspension was estimated to be 7.6 × 10^8^ cfu/ml. To an aliquot of 2.5 μl of this suspension, 12.25 μl of the direct qPCR components (2.7 μl of SW ver.) and 12.75 μl of the typical PCR master mix (*C. muytjensii omp*A specific primers) were added, and the direct qPCR was subsequently performed for 30 cycles.

Next, twenty 27.5-μl aliquots of the PCR products were combined into one portion and subjected to centrifugation at 3,000 × *g* for 10 min at 4 °C. A 5-μl aliquot of this supernatant was electrophoresed on a 2% agarose gel, followed by 1 × SYBR^®^ Gold nucleic acid gel staining (10,000 × concentrate in DMSO, Lonza Rockland, Inc., Rockland, ME, USA; hereafter, all gel staining was performed with SYBR Gold stain). After the residual supernatant was removed, 200 μl of physiological saline was subsequently added (estimated *C. muytjensii* cell number was 1.5 × 10^8^ cells/ml, considering the recovery loss due to a single centrifugation). The SYTO9 (Invitrogen) was added at a concentration of 1.5 μl/ml, followed by FCM measurements.

### Method to reveal that the reaction mechanism of the direct qPCR is similar to that of *in situ* PCR

Following the aforementioned fixation procedure, 500 μl of *C. muytjensii* cells (4.3 × 10^8^ cfu/ml) were cultured overnight and subjected to centrifugation, followed by removal of the supernatant, and 500 μl of fixation B (methanol/acetic acid = 3/1) was added to the pellet, followed by incubation overnight at 4 °C. As a control, a sample in which the fixation was not performed was obtained using the same volume of physiological saline instead of fixation agent. Next, the cells were washed 3 times with 500 μl of saline and suspended in 250 μl of saline. Because the recovery rate of bacterial cells to pellet after each centrifugation for washing was 80% (legend of Table 5) and centrifugation was conducted four times, the concentration of the *C. muytjensii* cells was estimated to be 3.5 × 10^8^ cells/ml.

To an aliquot of 2.5 μl of this suspension, 12.25 μl of the direct qPCR components (2.7 μl SW ver.) and 12.75 μl of the typical PCR master mix (total coliform-specific primers of 16 S_2751 F and 23 S_5201 R instead of *C. muytjensii omp*A primers) mentioned below were added to perform direct qPCR for retaining a fraction of the long PCR amplicons in *C. muytjensii* cells^20^. The forward primer 16 S_2751 F (5′-CTACAATGGCGCATACAAAGAGAAGCGACCT-3′) and reverse primer 23 S_5201 R (5′-CTTCTCCCGAAGTTACGGCACCA-3′) were used, and the principal elongated amplicon length was set at 2,451 bp^20^. The thermal cycle profile included 1 cycle at 4 °C for 3 min; 50 cycles at 95 °C for 15 sec, 60 °C for 20 sec, and 72 °C for 3 min, followed by 1 cycle at 95 °C for 3 min. Before or after the direct qPCR elongation, the 20 portions of the pre- or post-PCR products for *C. muytjensii* cells treated with a preliminary fixation B or no fixation were combined and subjected to centrifugation. The pellet was washed three times with 500 μl of saline to completely remove the long DNA amplicons potentially adsorbed onto the cell walls of *C. muytjensii*, and DNA purification was performed using the QuickGene SP kit DNA tissue to determine the presence of long DNA amplicons in the cells (Fuji Photo Film Co., Ltd., Tokyo, Japan).

### Comparing the direct qPCR with qPCR following crude DNA extraction or DNA purification methods to determine the detection limits for *Cronobacter* cells

From the *C. sakazakii* ATCC29544 and *C. muytjensii* ATCC51329 cultured overnight, for *Cronobacter* DNA standards, purified DNA free from RNA contamination was obtained following the typical SDS/protease treatment and phenol/chloroform extraction, followed by precipitation with ethanol together with RNase treatment to confirm the ratio of the optical density OD_260_ _nm_/OD_280_ _nm_ in a range of 1.8 to 2.0^16^,^31^. In contrast, after the aforementioned overnight cultures were washed with physiological saline, and the cells were serially diluted with SW. A 1-ml *Cronobacter* cell suspension was centrifuged, and the supernatant was removed. To each bacterial pellet, 12.25 μl of the direct qPCR components (2.7 μl SW ver.) and 12.75 μl of the typical PCR master mix (*omp*A), i.e., the direct qPCR master mix, were added for direct qPCR.

To examine the detection limits for *Cronobacter* cells by qPCR with purified DNA, DNA purification using the FastPure DNA Kit (Takara Bio) was performed for 1 ml bacterial suspension, and subsequently, an aliquot of 2.5 μl of purified DNA solution (200 μl) was included for qPCR according to the manufacturer’s instructions. Crude extraction through boiling 1 ml bacterial suspension for 10 min was performed, and subsequently, an aliquot of 2.5 μl of the supernatant obtained after centrifugation of the boiled suspension (1 ml) was included for qPCR.

As a control (qPCR standards using diluted purified DNA), 2.5 μl of the isolated DNA solutions was added to a new PCR tube at 6.5 fg to 650 pg/PCR tube, followed by the addition of the direct qPCR master mix. Because 1 cell of the bacteria was estimated to contain 5 fg of chromosomal DNA, each amount of DNA included in the PCR was identical to that of the associated bacterial cell number obtained from the centrifugation of each 1 ml bacterial suspension.

## Additional Information

**How to cite this article**: Soejima, T. *et al*. A novel mechanism for direct real-time polymerase chain reaction that does not require DNA isolation from prokaryotic cells. *Sci. Rep.* **6**, 28000; doi: 10.1038/srep28000 (2016).

## Supplementary Material

Supplementary Information

## Figures and Tables

**Table 1:** Direct qPCR elongation products from pellets or supernatants obtained from the centrifugation of *C. muytjensii* suspensions with or without PCR thermal cycling.

		Washing step	(Ct)*
*C. muytjensii* in saline (10^8^ cfu/ml)	No heat	Pellet (suspension)	16.4 ± 0.42†
		Supernatant	21.5 ± 0.00‡
	Heat following PCR thermal cycle	Pellet (suspension)	14.0 ± 0.49
		Supernatant	15.2 ± 0.21
*C. muytjensii* in direct qPCR components (10^8^ cfu/ml)	No heat	Pellet (suspension)	15.8 ± 1.48
		Supernatant	24.1 ± 1.84
	Heat following PCR thermal cycle	Pellet (suspension)	22.5 ± 0.49
		Supernatant	30.7 ± 0.35

^*^Direct qPCR measurements were performed in duplicate. The Ct values are presented as the means ± SD (n = 2).

^†^The live cell count of *C*. *muytjensii* in the Pellet was 7.6 ± 0.19 log_10_ cfu/ml on SPC agar.

^‡^The live cell count of *C. muytjensii* in the Supernatant was 5.7 ± 0.20 log_10_ cfu/ml on SPC agar.

**Table 2:** Effects of sample matrices on direct qPCR.

*C. muytjensii* live cells/PCR (cfu/PCR tube)	Physiological saline (Ct)	Direct qPCR components			
		No heat (Ct)		Heat following PCR thermal cycling (Ct)	
		Pellet†	Supernatant‡	Pellet†	Supernatant‡
2.5 × 10^6^	14.5 ± 0.49*	15.0 ± 0.65	14.9 ± 0.58	14.7 ± 0.64	14.6 ± 0.66
2.5 × 10^5^	17.3 ± 0.71	16.3 ± 0.41	16.4 ± 0.43	16.8 ± 0.63	16.7 ± 0.58
2.5 × 10^4^	20.9 ± 0.31	20.6 ± 0.47	20.5 ± 0.42	20.5 ± 0.58	20.3 ± 0.51
2.5 × 10^3^	23.8 ± 1.13	24.0 ± 0.72	24.2 ± 0.71	24.2 ± 0.75	24.0 ± 0.63

^*^Direct qPCR measurements were performed in duplicate. The Ct values are presented as the means ± SD (n = 2).

^†^The pellet was obtained through the centrifugation of the direct qPCR master mix with or without PCR thermal cycling. The freshly prepared direct qPCR master mix was added to the pellet to generate the matrices of labelled Pellet. These matrices were used to suspend *C. muytjensii*.

^‡^The supernatant was obtained through the centrifugation of direct qPCR master mix with or without PCR thermal cycling. These supernatants were used as the matrices labelled Supernatant to suspend **C. muytjensii**.

**Table 3:** FCM assay (SYTO9) for *C. muytjensii* with preliminary fixation of 4% paraformaldehyde before, during, or after the direct qPCR.

Fix: 0 cycle	Quadrant	Events	Quadrant rate in gated events (%)
	UL*	0 ± 0.0	0.00 ± 0.00
Total events: 5 × 10^4^	UR†	2 ± 0.7	0.01 ± 0.00
Gated events: 18994 ± 785.6	LL‡	539 ± 38.2	2.84 ± 0.20
	LR§	**18453 ± 748.1**	97.16 ± 3.94
**Fix: 15 cycles**			
	UL	0 ± 0.0	0.00 ± 0.00
Total events: 5 × 10^4^	UR	3 ± 2.1	0.02 ± 0.01
Gated events: 16648 ± 1030.3	LL	69 ± 13.4	0.41 ± 0.08
	LR	**16577 ± 1014.7**	99.58 ± 6.10
*Fix: 30 cycles*			
	UL	0 ± 0.0	0.00 ± 0.00
Total events: 5 × 10^4^	UR	7 ± 2.1	0.04 ± 0.01
Gated events: 16394 ± 971.6	LL	1602 ± 54.4	9.78 ± 0.33
	LR	**14786 ± 915.0**	90.19 ± 5.58

^*^UL presents Upper Left FCM quadrants, that is SYTO9 (−)/FL3-H (+).

^†^UR presents Upper Right FCM quadrants, that is SYTO9 (+)/FL3-H (+).

^‡^LL indicates Lower Left FCM quadrants, that is SYTO9 (−)/FL3-H (−).

^§^LR indicates Lower Right FCM quadrants, that is SYTO9 (+)/FL3-H (−).

**Table 4:** FCM assay (SYTO9) for *C. muytjensii* with preliminary fixation of (methanol/acetic acid = 3/1) mixture before, during, or after the direct qPCR.

Fix: 0 cycle	Quadrant	Events	Quadrant rate in gated events (%)
	UL*	0 ± 0.0	0.00 ± 0.00
Total events: 5 × 10^4^	UR†	3 ± 1.4	0.02 ± 0.01
Gated events: 16959 ± 1149.8	LL‡	5266 ± 174.7	31.09 ± 1.03
	LR§	**11691 ± 973.7**	68.90 ± 5.74
*Fix: 15 cycles*			
	UL	0 ± 0.0	0.00 ± 0.00
Total events: 5 × 10^4^	UR	2 ± 0.7	0.01 ± 0.00
Gated events: 16372 ± 610.2	LL	3671 ± 84.1	22.43 ± 0.51
	LR	**12700 ± 525.4**	77.57 ± 3.21
*Fix: 30 cycles*			
	UL	0 ± 0.0	0.00 ± 0.00
Total events: 5 × 10^4^	UR	4 ± 1.4	0.03 ± 0.01
Gated events: 14142 ± 553.7	LL	1394 ± 53.0	9.86 ± 0.37
	LR	**12844 ± 357.8**	90.82 ± 2.53

^*^UL presents Upper Left FCM quadrants, that is SYTO9 (−)/FL3-H (+).

^†^UR presents Upper Right FCM quadrants, that is SYTO9 (+)/FL3-H (+).

^‡^LL indicates Lower Left FCM quadrants, that is SYTO9 (−)/FL3-H (−).

^§^LR indicates Lower Right FCM quadrants, that is SYTO9 (+)/FL3-H (−).

**Table 5:** Comparison between direct qPCR without any DNA extraction and qPCR with crude or purified DNA extraction.

Bacteria conc. (cfu/ml)		1.6 × 10^5^	1.6 × 10^4^	1.6 × 10^3^	1.6 × 10^2^	1.6 × 10^1^	1.6 × 10^0^
*C. sakazakii* ATCC29544	DNA*	22.1 ± 0.78¶	25.2 ± 0.42	28.6 ± 0.85	32.3 ± 0.99	36.1 ± 1.13	41.9 ± 1.56
	Cell†	21.6 ± 0.44	25.0 ± 0.26	28.9 ± 0.78	32.2 ± 0.75	35.6 ± 0.92	40.6 ± 1.27
	Clean-up‡	26.2 ± 0.63	29.5 ± 0.55	35.3 ± 1.03	ND × 2	ND × 2	ND × 2
	Boil‡	28.8 ± 0.69	32.3 ± 0.61	38.2 ± 1.14	ND × 2	ND × 2	ND × 2
*C. muytjensii* ATCC51329	DNA*	19.9 ± 0.71¶	23.4 ± 0.35	26.0 ± 0.85	30.7 ± 0.85	35.4 ± 1.13	40.2 ± 1.70
	Cell†	18.3 ± 0.52	21.8 ± 0.51	24.5 ± 0.69	29.4 ± 0.84	34.8 ± 0.86	39.6 ± 1.03
	Clean-up‡	23.3 ± 0.62	27.8 ± 0.54	31.6 ± 0.77	37.1 ± 1.15	ND × 2	ND × 2
	Boil‡	25.4 ± 0.71	28.9 ± 0.73	33.9 ± 0.89	ND × 2	ND × 2	ND × 2

^*^DNA represents qPCR using serially diluted purified chromosomal DNA.

^†^Cell represents direct qPCR using *Cronobacter* cells (all of the pellet obtained through centrifugation of 1-ml suspension) without DNA extraction. Through centrifugation of 1.6 × 10^5^ cfu/ml of bacterial suspension (1-ml), SPC counts revealed that 1.3 × 10^5^ cfu of *Cronobacter* cells was recovered in the pellet.

^‡^Clean-up indicates DNA purification using FastPure DNA Kit (Takara Bio) for 1-ml suspension; subsequently, an aliquot of 5 μl of purified DNA solution (200 μl) was used for qPCR. Boil indicates the crude extraction of boiling 1-ml suspension for 10 min; subsequently, an aliquot of 5 μl of the supernatant obtained through centrifugation of the boiled suspension (1-ml) was used for qPCR.

^¶^The estimated chromosomal DNA amounts in 1.3 × 10^5^ cfu of *C. sakazakii*, using the equation,1 cfu = 5 fg of chromosomal DNA, were used for qPCR measurements (n = 2), and the Ct values are presented as the means ± SD (n = 2).
