# Supplementary material for: A novel mechanism for direct real-time polymerase chain reaction that does not require DNA isolation from prokaryotic cells
Source: Sci Rep. 2016 Jun 23;6:28000. doi: 10.1038/srep28000 (PMC4917819; doi:10.1038/srep28000)
Supplement: Supplementary Information [file srep28000-s1.pdf]

SUPPLEMENTARY INFORMATION

A novel mechanism for direct real-time polymerase chain reaction that does not require DNA isolation from prokaryotic cells

Takashi Soejima[1]*, Jin-zhong Xiao[2] & Fumiaki Abe[1]

[1]Functional Food Ingredients, Food Ingredients & Technology Institute, [2]Next Generation Science Institute, Morinaga Milk Industry Co., Ltd. 5-1-83, Higashihara, Zama, Kanagawa, 252-8583, Japan.

* To whom correspondence should be addressed: Takashi Soejima; E-mail:

t_soezim@morinagamilk.co.jp; Tel: 81-46-252-3066; Fax: 81-46-252-3017

Methods

Bacterial inoculation of pasteurized milk. The live cell suspension stock was diluted 10-fold with marketed pasteurized cow's milk (hereafter, milk) to prepare a live cell milk suspension of *C. muytjensii*. Moreover, the dead cell suspension stock was diluted 10-fold with milk to prepare a dead cell milk suspension of *C. muytjensii*.

Ethidium monoazide (EMA) treatment followed by irradiation with visible light. EMA (Sigma, St. Louis, MO, USA) was dissolved at a concentration of 1000 µg/ml using SW and sterilized with a 0.20-µm filter (Minisart Plus, Sartorius AG, Göttingen, Germany) to prepare a stock solution that was stored at -20 °C under light shielding[1]. EMA was used to suppress the PCR elongation resulting from the dead background bacteria originally contaminating the pasteurized milk[1].

A volume of 10 µl of the EMA stock solution (1000 µg/ml) was added to the live and dead *C. muytjensii* cell suspensions in physiological saline (1 ml), and the suspensions were stored at 4 °C for 10 min under light shielding[1]. After the suspensions were placed at a distance of 20 cm from a visible light source (100 V, PRF 500 W, Flood Eye, Iwasaki Electric Co., Ltd., Tokyo, Japan), the cells were irradiated with visible light for 5 min on ice[1]. The EMA-treated samples were subjected to refrigerated centrifugation at 15,000 × *g* for 10 min at 4 °C, the supernatants were removed, and 1 ml of physiological saline was added to the pellet for washing. A volume of 10 µl SW was added to the bacterial pellets, and 5 µl (test sample) was applied for PCR elongation without DNA isolation (direct qPCR), as presented in the main text.

Similarly, the live and dead *C. muytjensii* cell milk suspensions (1 ml) were treated with EMA, followed by visible light irradiation, as shown below. Each live and dead cell milk suspension (1 ml) was centrifuged at 15,000 × *g* for 10 min at 4 °C, the supernatant was removed, and 1 ml of physiological saline was subsequently added. Next, 3 µl protease (resulting from the *Bacillus* bacterium, Sigma) was added, and the sample was incubated at 37 °C for 1 h, followed by centrifugation at 15,000 × *g* for 10 min at 4 °C[2]. The supernatant was removed, 1 ml of physiological saline was added, and 10 µl of EMA solution (1000 µg/ml) was subsequently added in the dark. Hereafter, the EMA treatment procedure was the same as that of the EMA-treated live cell and dead *C. muytjensii* cell suspensions in physiological saline (1 ml).

Preparation of the direct qPCR master mix to circumvent the suppression of PCR due to potent PCR inhibitors present in milk. An agent comprising trisodium citrate dehydrate (TSC, Kanto Kagaku, Tokyo, Japan) and magnesium chloride hexahydrate (Nakalai-Tesque, Kyoto, Japan) and further containing one or more compounds selected from bovine serum albumin (BSA, Sigma), dextran (low molecule, M.W. 50,000 to 70,000, Nakalai-Tesque), T4 gene protein 32 (gp32, Nippon Gene, Toyama, Japan), sodium lauryl sulphate (SDS, Nakalai-Tesque), polyoxyethylene (10) cetyl ether (Brij56) (Sigma), and egg white lysozyme (Wako Pure Chemical Industries, Osaka, Japan) was added to 5 µl of the test sample for direct qPCR elongation. The detailed compositions (abbreviated as direct qPCR components) are presented below:

Composition 1: 5 µl 2 % BSA, 1 µl 50 mM TSC, 1.5 µl 100 mM $MgCl_2$, and 1 µl 0.05 % SDS; Composition 2: 5 µl 2 % BSA, 1 µl 50 mM TSC, and 1.5 µl 100 mM $MgCl_2$; Composition 3: 2.5 µl 20 % Dextran, 1 µl 50 mM TSC, and 1.5 µl 100 mM $MgCl_2$; Composition 4: 5 µl 0.1 % gp32, 1 µl 50 mM TSC, and 1.5 µl 100 mM $MgCl_2$; Composition 5: 5 µl 2 % BSA, 1 µl 50 mM TSC, 1.5 µl 100 mM $MgCl_2$, and 12.6 µl 4 % Brij56; Composition 6: 5 µl 2 % BSA, 1 µl 50 mM TSC, 1.5 µl 100 mM $MgCl_2$, and 1.0 µl 500 µg/ml egg white lysozyme; Composition 7: 5 µl 2 % BSA, 1 µl 50 mM TSC, 1.5 µl 100 mM $MgCl_2$, 1 µl 0.05 % SDS, 12.6 µl 4 % Brij56, and 1.0 µl 500 µg/ml egg white lysozyme; Composition 8: 5 µl 2 % BSA, 1 µl 50 mM TSC, 1.5 µl 100 mM $MgCl_2$, 12.6 µl 4 % Brij56, and 1.0 µl 500 µg/ml egg white lysozyme; Composition 9: 5 µl 2 % BSA; Composition 10: typical PCR master-mix alone, comprising the below-mentioned reagents: Primer F: universal forward primer 16S_10F for 16S rRNA gene amplification (5'-AGTTTGATCCTGGCTC-3') and Primer R: universal reverse primer 16S_1500R for 16S rRNA gene amplification (5'-GGCTACCTTGTTACGA-3'), both of which were used as PCR primers. The target gene by the primers was a long DNA (1,491_bp)[3]. The typical PCR master-mix consisted of 4 µl 10 pmol/µl Primer F, 4 µl 10 pmol/µl Primer R, 0.5 µl 5U/µl Ex-Taq (Takara-Bio, Ohtsu, Japan) that contained 0.5 % Tween20, 0.5 % octylphenolpoly(ethyleneglycolether)$_n$ (Nonidet P-40), and 50 % glycerol, 5 µl of 10 × Ex-Taq Buffer (Takara-Bio), 4 µl dNTP mixture (2 mM each; Toyobo), 8 µl of 10 × SYBR Green I Nucleic Acid Gel Stain (Lonza Rockland, Inc., Rockland, ME, USA), and SW required to obtain the total volume of 55 µl, including 5 µl of the test sample and the aforementioned direct qPCR components. Real-time PCR was performed according to the following PCR thermal cycle profile using the real-time PCR machine (iCycleriQ, Bio-Rad, Hercules, CA, USA). The thermal cycle profile was 1 cycle at 4 °C for 3 min (1 cycle), 1 cycle at 94 °C for 30 sec, 50 cycles at 94 °C for 20 sec; 55 °C for 30 sec; 72 °C for 1 min and 30 sec, followed by 1 cycle at 95 ° C for 3 min. Subsequently, for the melt analysis of the PCR amplicon, the temperature was raised at intervals of 0.1 °C from 60 °C, each temperature was maintained for 8 sec, and this increasing step was repeated 350 times in total up to the final temperature of 95 °C.

Results and Discussion

Screening of the direct qPCR components to specifically detect live *C. muytjensii* suspended in physiological saline or milk

Supplementary Table 1 shows the specific detection of live *C. muytjensii* in physiological saline or milk subjected to ethidium monoazide (EMA) treatment[1,2,4,5], followed by direct qPCR without DNA extraction. Compositions 1 to 10 led to the specific detection of live *C. muytjensii* in physiological saline. However, for Composition 10, the Ct values with and without EMA for live cells and those of non-treated dead cells were much higher (i.e., more poorly detected) than those of the associated cells from Compositions 1-9. Furthermore, Compositions 1-9 succeeded in specifically detecting live *C. muytjensii* in pasteurized milk, but only Composition 10 (i.e., typical PCR master mix alone) did not facilitate elongation in live and dead cells,

thus implying PCR suppression through PCR inhibitors present in the milk. In relation to the Ct values of direct qPCR, particularly when targeting good PCR elongation with the live *C. muytjensii* cells (with and without EMA) in milk containing many PCR inhibitors, the Ct values of the live *C. muytjensii* cells for Compositions 7 and 8 were the lowest among all of the Compositions (1-10). Briefly, in the presence of numerous PCR inhibitors, Compositions 7 and 8 facilitated the highly effective elongation of the targeted gene in the bacterial cells.

References

1. Soejima, T. *et al*. Method to detect only live bacteria during PCR amplification. *J. Clin. Microbiol.* **46**, 2305-2313 (2008).
2. Soejima, T., Minami, J.-I., Yaeshima, T. & Iwatsuki, K. An advanced PCR method for the specific detection of viable total coliform bacteria in pasteurized milk. *Appl. Microbiol. Biotechnol*. **5**, 485-497 (2012).
3. Soejima, T., Schlitt-Dittrich, F. & Yoshida, S-I. Polymerase chain reaction amplification length-dependent ethidium monoazide suppression power for heat-killed cells of *Enterobacteriaceae*. *Anal. Biochem.* **418**, 286-294 (2011). .
4. Rudi, K., Moen, B., Dromtorp, S.M. & Holck, A.L. Use of ethidium monoazide and PCR in combination for quantification of viable and dead cells in complex samples. *Appl. Environ. Microbiol.* **71**, 1018–1024 (2005).
5. Nocker, A. & Camper, A.K. Selective removal of DNA from dead cells of mixed bacterial communities by use of ethidium monoazide. *Appl. Environ. Microbiol.* **72**, 1997–2004 (2006).

Supplementary Table captions

Supplementary Table 1 | Distinguishing between live and dead *C. muytjensii* in physiological saline or pasteurized milk after EMA treatment, followed by direct qPCR.
* No. 10: a typical PCR master mix; Dex: dextran; GP32: T4 gene 32 protein; TSC: trisodium citrate dehydrate; Lyso: lysozyme.
[†](+): addition; (-): no addition.
[‡]EMA (-): no EMA treatment. EMA (+): 10 µg/ml for 10 min and light-irradiation for 5 min.
[§] Direct qPCR was performed in duplicate, and the Ct values are presented as the means ± SD (n = 2). ND: no elongation (n = 2).

Supplementary Table 1 | Distinguishing between live and dead *C. muytjensii* in physiological saline or pasteurized milk due to EMA treatment followed by the direct qPCR

No.	Direct qPCR components*								*C. muytjensii* in physiological saline (8.95 ± 0.01 log_{10}cfu or cells/ml)				*C. muytjensii* in pasteurized milk (8.95 ± 0.01 log_{10}cfu or cells/ml)			
									Live (Ct)		Heat-killed (Ct)		Live (Ct)		Heat-killed (Ct)	
	BSA	Dex	GP32	TSC	$MgCl_2$	SDS	Brij56	Lyso	EMA (-)[‡]	EMA (+)[‡]	EMA (-)	EMA (+)	EMA (-)	EMA (+)	EMA (-)	EMA (+)
1	+[†]	-[†]	-	+	+	+	-	-	17.3 ±0.92[§]	21.1 ± 1.48	20.1 ± 1.48	ND[§]	32.1 ± 0.78	35.2 ± 0.92	35.2 ± 1.13	ND[§]
2	+	-	-	+	+	-	-	-	17.1 ± 0.57	20.2 ± 1.41	20.3 ± 1.13	ND	31.0 ± 1.41	34.4 ±1.63	34.9 ± 0.99	ND
3	-	+	-	+	+	-	-	-	17.3 ± 0.71	20.1 ± 0.78	20.4 ± 1.20	ND	32.2 ± 0.71	34.1 ± 1.41	35.2 ± 1.20	ND
4	-	-	+	+	+	-	-	-	18.2 ± 0.49	20.3 ± 1.77	20.2 ± 1.77	ND	33.3 ± 0.64	35.1 ± 0.57	35.4 ± 1.77	ND
5	+	-	-	+	+	-	+	-	16.0 ± 0.85	19.4 ± 0.92	19.1 ± 0.78	ND	25.3 ± 1.20	27.1 ± 0.78	27.3 ± 1.47	ND
6	+	-	-	+	+	-	-	+	17.1 ± 1.13	19.3 ± 0.57	19.1 ± 0.57	ND	28.1 ± 0.92	31.4 ± 0.78	31.8 ± 0.92	ND
7	+	-	-	+	+	+	+	+	15.2 ±0.64	18.2 ±1.20	18.1 ± 0.78	ND	21.9 ± 0.78	23.7 ± 0.49	24.3 ± 1.48	ND
8	+	-	-	+	+	-	+	+	16.1 ± 0.42	19.1 ± 1.27	19.0 ± 1.20	ND	21.7 ± 0.85	23.7 ± 0.78	24.1 ± 1.20	ND
9	+	-	-	-	-	-	-	-	18.2 ± 0.78	21.0 ± 1.13	21.0 ± 1.27	ND	33.8 ± 1.13	37.2 ± 1.41	37.7 ± 1.63	ND
10	-	-	-	-	-	-	-	-	25.1 ± 0.71	27.9 ±1.13	28.2 ± 1.84	ND	ND	ND	ND	ND

* No. 10: a typical PCR mastermix; Dex: dextran; GP32: T4 gene 32 protein; TSC: trisodium citrate dehydrate; Lyso: lysozyme.

[†](+): addition; (-): no addition.

[‡]EMA (-): no EMA treatment. EMA (+): 10 µg/ml for 10 min and light-irradiation for 5 min.

[§]Direct qPCR were performed in duplicate, and Ct values are presented as the means ± SD (n = 2). ND: no elongation (n = 2).
